# An increase in cancer stem cell population after primary systemic therapy is a poor prognostic factor in breast cancer

**DOI:** 10.1038/bjc.2011.159

**Published:** 2011-05-10

**Authors:** H E Lee, J H Kim, Y J Kim, S Y Choi, S-W Kim, E Kang, I Y Chung, I A Kim, E J Kim, Y Choi, H S Ryu, S Y Park

**Affiliations:** 1 Department of Pathology, Seoul National University Hospital, 28 Yeongon-dong, Jongno-gu, Seoul 110-744, Korea; 2 Department of Internal Medicine, Seoul National University College of Medicine, 28 Yeongon-dong, Jongno-gu, Seoul 110-799, Korea; 3 Department of Surgery, Seoul National University College of Medicine, 28 Yeongon-dong, Jongno-gu, Seoul 110-799, Korea; 4 Breast Care Center, Seoul National University Bundang Hospital, 300 Gumi-dong, Bundang-gu, Seongnam-si, Gyeonggi 463-707, Korea; 5 Department of Radiation Oncology, Seoul National University College of Medicine, 28 Yeongon-dong, Jongno-gu, Seoul 110-799, Korea; 6 Department of Pathology, Seoul National University Bundang Hospital, 300 Gumi-dong, Bundang-gu, Seongnam-si, Gyeonggi 463-707, Korea; 7 Department of Pathology, Seoul National University College of Medicine, 28 Yeongon-dong, Jongno-gu, Seoul 110-799, Korea

**Keywords:** breast cancer, cancer stem cells, primary systemic therapy, chemoresistance

## Abstract

**Background:**

The cancer stem cell (CSC) hypothesis has important clinical implications for cancer therapeutics because of the proposed role of CSCs in chemoresistance. The aim of this study was to investigate changes in the CSC populations before and after primary systemic therapy (PST) and their prognostic role in human breast cancer.

**Methods:**

Paired samples (before and after PST) of breast cancer tissue were obtained from clinical stage II or III patients (*n*=92) undergoing PST with the regimen of doxorubicin plus docetaxel (AD) (*n*=50) or doxorubicin plus cyclophosphamide (AC) (*n*=42) and subsequent breast resection. The proportions of putative CSCs with CD44+/CD24− or aldehyde dehydrogenase 1+ (ALDH1+) phenotypes were determined by immunohistochemistry.

**Results:**

A higher proportion of CD44+/CD24− tumour cells and ALDH1 positivity in pre-chemotherapy tissue was correlated with higher histologic grade, oestrogen receptor (ER) negativity, high Ki-67 proliferation index and basal-like subtype of breast cancer. Aldehyde dehydrogenase 1 positivity in pre-chemotherapy biopsy was also associated with a higher rate of pathologic complete response following PST. In comparisons of putative CSC populations before and after PST, the proportions of CD44+/CD24− and ALDH1+ tumour cells were significantly increased after PST. The cases with increased CD44+/CD24− tumour cell populations after PST showed high Ki-67 proliferation index in post-chemotherapy specimens and those with increased ALDH1+ tumour cell population after PST were associated with ER negativity and p53 overexpression. Furthermore, cases showing such an increase had significantly shorter disease-free survival time than those with no change or a reduced number of CSCs, and the survival difference was most notable with regard to the changes of ALDH1+ tumour cell population in the patients who received AC regimen.

**Conclusion:**

The present study provides the clinical evidence that the putative CSCs in breast cancer are chemoresistant and are associated with tumour progression, emphasising the need for targeting of CSCs in the breast cancer therapeutics.

Primary (neoadjuvant) systemic therapy (PST) is currently considered the standard care for locally advanced breast cancers (Buzdar, 2007; Gralow *et al*, 2008). Primary systemic therapy permits breast-conserving surgery and treats clinically undetectable micrometastatic disease before surgery. In addition, the degree of response to PST can be used as a prognostic indicator and pathologic complete response (pCR) is now recognised as an independent prognostic factor of patients with locally advanced breast cancer, who had received anthracycline-based or anthracycline- and taxane-based chemotherapy (Wolmark *et al*, 2001; Bear *et al*, 2006; Guarneri *et al*, 2006; Buzdar, 2007; Symmans *et al*, 2007; Chavez-MacGregor *et al*, 2010). On the other hand, drug resistance has become the major cause of treatment failure and is largely responsible for breast cancer mortality (Dean *et al*, 2005; Kakarala and Wicha, 2008; Morrison *et al*, 2008). Overcoming resistance to chemotherapeutic agents would represent a major advance in the clinical management of breast cancer.

There is accumulating evidence to support the cancer stem cell (CSC) hypothesis, which states that cancers are driven by a small population of stem cells that are capable of self-renewal and give rise to multipotent progenitor cells, and eventually, differentiate into all cell types within the tumour (Smalley and Ashworth, 2003; Clarke *et al*, 2006; Campbell and Polyak, 2007; Visvader and Lindeman, 2008; Nakshatri *et al*, 2009). Cancer stem cells may originate by malignant transformation of normal stem cells that have accumulated genetic and epigenetic modifications over time (Smalley and Ashworth, 2003; Dean *et al*, 2005; Visvader and Lindeman, 2008). Alternatively, they may arise from restricted progenitors of more differentiated cells that have acquired self-renewing capacity through multiple genetic and epigenetic alterations (Smalley and Ashworth, 2003; Dean *et al*, 2005; Visvader and Lindeman, 2008).

Cancer stem cells were first identified in acute myeloid leukaemia (Lapidot *et al*, 1994), and recently, have also been documented in various solid tumours, including those of breast (Al-Hajj *et al*, 2003), brain (Singh *et al*, 2004), lung (Kim *et al*, 2005), colon (O’Brien *et al*, 2007), prostate (Collins *et al*, 2005) and pancreas (Li *et al*, 2007). In the case of breast tumours, Al-Hajj *et al* (2003) were the first to isolate a highly tumourigenic subpopulation of tumour cells with CD44^+^/CD24^−/low^/Lineage^−^ phenotype. Subsequently, Ginestier *et al* (2007) presented evidence that aldehyde dehydrogenase 1 (ALDH1) is a marker of stem/progenitor cells of the normal and malignant human breast. They demonstrated the tumourigenicity of ALDH1-positive cells by injection of ALDEFLUOR-positive population into NOD/SCID mice and detected ALDH1 expressing tumour cells in breast carcinoma tissue by immunohistochemistry. However, it is also known that none of these CSC markers is expressed exclusively by stem cells, and a considerable number of cells that express these markers are not stem cells, resulting in phenotypic heterogeneity within putative CSC populations (Clarke *et al*, 2006; Visvader and Lindeman, 2008).

The CSC hypothesis has important clinical implications for cancer therapeutics because of the suggested role of CSCs in chemoresistance (Kakarala and Wicha, 2008). There is increasing evidence that CSCs are naturally resistant to chemotherapy on account of their quiescence, more efficient DNA repair, resistance to apoptosis and expression of drug-resistance proteins, such as ATP-binding cassette transporters (ABCG2 and ABCG5) and multidrug-resistance protein 1 transporters (Dean *et al*, 2005). If this is correct, a small population of chemoresistant CSCs may resist killing by conventional chemotherapy, whereas majority of tumour cells, which are differentiated cells that lack ‘stemness’, may be killed. The tumour could, therefore, regrow after chemotherapy because of the capacity for self-renewal of these CSCs. However, there have been few reports of chemoresistance of CSCs in human breast tissue (Li *et al*, 2008; Tanei *et al*, 2009), although several *in vitro* studies have indicated it (Liu *et al*, 2006; Ghods *et al*, 2007; Yu *et al*, 2007; Fillmore and Kuperwasser, 2008).

The aim of this study was to investigate changes in CSC populations before and after PST and their prognostic role in human breast cancer. We determined the proportions of putative CSCs with CD44+/CD24− or ALDH1+ phenotypes using immunohistochemistry in pre- and post-chemotherapy breast cancer tissue, and analysed the association between the changes of putative CSC population after PST and clinicopathologic parameters, including patients’ survival.

## Patients and methods

### Patients and specimens

Ninety-two patients with clinical stage II or III breast cancer (by AJCC 7th), who received surgical resection after PST in Seoul National University Bundang Hospital from May 2003 to February 2009 were enrolled. All patients were diagnosed to have invasive breast carcinoma by core needle biopsy, and were started on PST. Fifty patients were treated with doxorubicin plus docetaxel (AD) regimen containing 50 mg m^–2^ doxorubicin i.v. on day 1 and 75 mg m^–2^ docetaxel i.v. on day 1 every 3 weeks for three to six cycles (mean, five cycles). Forty-two patients received doxorubicin plus cyclophosphamide (AC) regimen containing 60 mg m^–2^ doxorubicin i.v. on day 1 and 600 mg m^–2^ cyclophosphamide i.v. on day 1 every 3 weeks for four cycles. Formalin-fixed and paraffin-embedded paired tissue samples of pre-chemotherapy biopsy and post-chemotherapy surgical resection were collected from each patient. None had received radiation therapy preoperatively. All were female, with a median age of 46 (range, 28–70). Clinicopathologic information was obtained by reviewing medical records, pathology reports and H&E-stained sections. The following histopathologic variables were determined: histologic subtype, Bloom-Richardson histologic grade, T stage, N stage, oestrogen receptor (ER), progesterone receptor (PR), HER2 status, Ki-67 proliferation index and P53 expression. Pathological complete response following PST was defined as complete disappearance of all invasive cancer or only residual ductal carcinoma *in situ*. The duration of disease-free survival (DFS) was defined as the time between the start of PST and first recurrence/metastasis or death. All cases were independently reviewed by two breast pathologists (SYP and HEL). This study was approved by the Institutional Review Board of Seoul National University Bundang Hospital (protocol # B-1008-110-301), waiving the requirement for informed consent for the study.

### Immunohistochemical staining

Formalin-fixed and paraffin-embedded sections were dewaxed in xylene, rehydrated through graded alcohol and placed in an endogenous peroxide block for 15 min. Antigen retrieval was carried out by microwave in 10 mM citrate buffer. Nonspecific staining was blocked by treating sections with 10% goat serum in phosphate-buffered saline (pH 6.0) for 10 min. Immunohistochemical staining was carried out in a DAKO Autostainer Plus (Dako, Glostrup, Denmark) using an LSAB detection kit (Dako). The following primary antibodies were used: CD44 (1 : 200; clone 156-3C11; Neomarkers, Fremont, CA, USA), CD24 (1 : 100; clone SN3b; Neomarkers), ALDH1 (1 : 100; clone 44; BD Biosciences, Franklin Lakes, NJ, USA), cytokeratin 5/6 (CK5/6) (1 : 50; clone D5/16 B4; Dako) and epidermal growth factor receptor (EGFR) (EGFR pharmDx, Dako). The proportions of each antibody-positive tumour cells were counted semiquantitatively.

### Double immunohistochemical staining for CD44 and CD24

Double immunostaining with antibodies to detect CD44 and CD24 was performed with EnVision G∣2 Doublestain System Rabbit/Mouse (diaminobenzidine+ (DAB+)/Permanent Red) (Dako, Carpinteria, CA, USA) according to the manufacturer's instructions. CD44 was detected with DAB and CD24 with Permanent Red. We confirmed the accuracy of the double immunostaining by comparing it with single immunostaining for CD44 and CD24, separately. The proportions of CD44+/CD24− tumour cells was counted semiquantitatively and scored in 5% increments.

### Breast cancer subtypes

Although intrinsic subtypes of breast cancer were originally defined by gene expression profiling using DNA microarrays, most archival formalin-fixed paraffin-embedded samples are not amenable to cDNA microarray and subsequent studies revealed that subtypes can be accurately determined using immunohistochemical as a surrogate for molecular classification (Nielsen *et al*, 2004; Carey *et al*, 2006). Thus, breast cancer subtypes were defined as previously with minor modifications (Nielsen *et al*, 2004; Carey *et al*, 2006). Subtype definitions in this study were as follows: luminal A (ER+ and/or PR+, HER2−), luminal B (ER+ and/or PR+, HER2+), HER2+ (ER−, PR−, HER2+), basal-like (ER−, PR−, HER2−, CK5/6+ and/or EGFR+) and unclassified (negative for all markers).

With regard to immunostaining for ER and PR, cases with 10% or more positive staining were grouped as positive. Expression of EGFR and HER2 was scored as follows: 0, no staining; 1+, weak and incomplete membranous staining in ⩾10% of the tumour cells; 2+, weak to moderate, complete membranous staining in ⩾10% of the tumour cells and 3+, strong, complete membranous staining in ⩾30% of the tumour cells. Any positive staining was regarded as positive for EGFR, and 3+ on immunohistochemistry or presence of gene amplification on fluorescence *in situ* hybridisation was considered HER2 positive. For CK5/6, cases with any degree of positive staining were grouped as positive.

### Statistical analyses

Non-continuous variables were compared using the *χ*^2^-test or Fisher’s exact (two-sided) test and continuous variables using the Mann–Whitney *U-*test. Univariate and multivariate logistic regression models were used to identify predictive factors of pCR following PST. Changes of the CD44+/CD24− and ALDH1+ tumour cell populations before *vs* after PST were evaluated by Wilcoxon's signed-rank test. Survival curves were estimated using the Kaplan–Meier product-limit method, and the significances of differences between survival curves were determined using the log-rank test. The covariates, which were statistically significant in the univariate analysis, were then included in the multivariate analysis using Cox proportional hazards regression model; the hazard ratio (HR) and its 95% confidence interval (CI) were assessed for each factor. All statistical analyses were conducted using SPSS statistics 17.0 (SPSS Inc., Chicago, IL, USA), and *P-*values of ⩽0.05 were considered statistically significant.

## Results

### Detection of CD44+/CD24− and ALDH1+ tumour cells in pre-chemotherapy biopsy tissue

In 92 pre-chemotherapy biopsy tissues, median values of CD44+/CD24− and ALDH1+ tumour cell proportions were 5% (range, 0–95%) and 0% (0–80%), respectively. When ALDH1 immunostaining was classified into negative (ALDH1+ tumour cells <5%), 1+ (⩾5%, <10%,), 2+ (⩾10%, <50%) and 3+ (>50%) groups, there were 80 (87.0%), 3 (3.3%), 8 (8.7%) and 1 (1.1%) cases assigned to the negative, 1+, 2+ and 3+ groups, respectively. We further classified the cases into negative (<5% *n*=80) and positive (⩾5% *n*=12) groups for ALDH1 and found that the ALDH1-positive group contained a higher proportion of CD44+/CD24− tumour cells than the ALDH1-negative group (median (interquartile range), 45% (20–70%) *vs* 5% (1–30%); *P*=0.003).

### Association between CD44+/CD24− and ALDH1+ tumour cell populations in pre-chemotherapy tissue and clinicopathologic characteristics

We analysed the relationship between CD44/CD24 and ALDH1 expression status in pre-chemotherapy tissue and various clinicopathologic parameters. A higher proportion of CD44+/CD24− tumour cells and ALDH1 positivity was associated with higher histologic grade (*P*=0.002 and *P*=0.007, respectively) and ER negativity of tumour (*P*<0.001 and *P*<0.001, respectively). Neither clinical T nor clinical node stage was related to CD44/CD24 or ALDH1 status. The differences in CD44+/CD24− tumour cell proportions and ALDH1 positivity between the subtypes of breast cancer were statistically significant. Specifically, the basal-like subtype had the highest proportion of CD44+/CD24− tumour cells and the highest frequency of ALDH1 positivity among the subtypes. In addition, Ki-67 proliferation index was positively correlated with ALDH1 status (*P*=0.020) (Table 1). CD44/CD24 and ALDH1 expression status in pre-chemotherapy specimens was not associated with DFS (data not shown).

### CD44+/CD24− and ALDH1+ tumour cell population as a predictive factor of pCR

**Figure 1:**
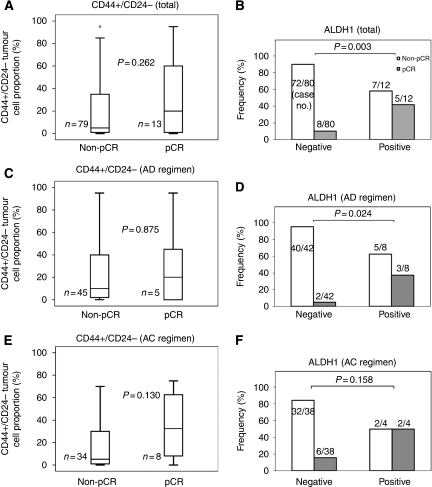
Association of CD44+/CD24− and ALDH1+ status with pathologic complete responses (pCR) following primary systemic therapy in total patients (**A** and **B**) and in the subgroups by the primary systemic therapy regimen (**C**–**F**). The group showing pCR tended to have a higher proportion of CD44+/CD24− tumour cells than the non-pCR group in total patients (**A**) and in the subgroup treated with AC regimen (**E**). The ALDH1 positive group had a significantly higher likelihood of pCR than the ALDH-1-negative group in total (**B**). This association was also found irrespective of primary systemic therapy regimen (AD or AC regimen), although a statistical significance was reached only for AD regimen (**D** and **F**). AD=doxorubicin plus docetaxel; AC=doxorubicin plus cyclophosphamide.

Thirteen (13.7%) of the 92 patients, that is 5 (10.0%) of 50 patients who were treated with AD regimen and 8 (19.0%) of 42 patients who received AC regimen, achieved a pCR after PST and there was no statistical difference in pCR rate according to PST regimen. Aldehyde dehydrogenase 1 positivity of pre-chemotherapy tissue was significantly correlated with a higher rate of pCR after PST (10 *vs* 42% *P*=0.003; Figure 1B). This association was also found according to PST regimen (AD or AC regimen), although a statistical significance was reached only for AD regimen (*P*=0.024) (Figures 1D and F). The pCR group also showed a tendency to have a higher proportion of CD44+/CD24− tumour cells than the non-pCR group not showing pCR in total patients (*P*=0.262) and in the subgroup receiving AC regimen (*P*=0.130) (Figures 1A, C and E).

To determine whether CD44+/CD24− tumour cell proportion and ALDH1 positivity were predictive factors of pCR, we performed logistic regression analyses. In univariate analysis, ALDH1 positivity and high histologic grade were found to be significantly associated with pCR (*P*=0.007 and 0.037, respectively). However, in multivariate analysis, they did not remain as independent predictive factors. CD44+/CD24− tumour cell proportion was not a significant predictive factor of pCR (Table 2).

### Changes of CD44+/CD24− and ALDH1+ tumour cell populations before and after PST and their relationships with DFS

**Figure 2:**
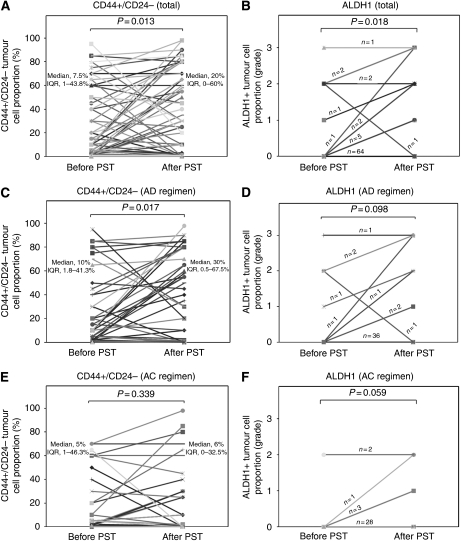
Changes of putative CSC populations after primary systemic therapy (PST) in the cases not achieving pathologic complete response. CD44+/CD24− tumour cell proportions (**A**) and ALDH1+ tumour cell grades (**B**) increased significantly after PST in total. Within the subgroup receiving AD regimen (**C** and **D**), CD44+/CD24− tumour cell proportions increased significantly (**C**) and the grades of ALDH1+ tumour cells showed a tendency to increase after PST (**D**). In the subgroup treated with AC regimen (**E** and **F**), only grades of ALDH1+ tumour cells increased after PST with a marginal significance (**F**). AD=doxorubicin plus docetaxel; AC=doxorubicin plus cyclophosphamide.

**Figure 3:**
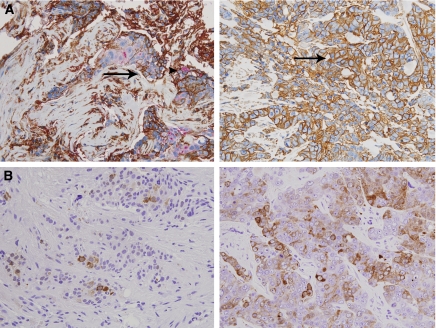
A representative example of the changes in putative CSC populations after primary systemic therapy. Both CD44+/CD24− (**A**) and ALDH1+ (**B**) tumour cells were increased after PST in this case. An arrow head, a CD44+/CD24+ cell; arrows, CD44+/CD24− cells (original magnifications, × 400).

We assessed changes of CD44+/CD24− and ALDH1+ tumour cell populations before and after PST in the 79 patients who did not achieve pCR. Both CD44+/CD24− tumour cell proportions and grades of ALDH1+ tumour cells significantly increased after PST (*P*=0.013 and *P*=0.018, respectively) (Figures 2A, B and 3). Within the subgroup receiving AD regimen, CD44+/CD24− tumour cell proportions also increased significantly (*P*=0.017) and the grades of ALDH1+ tumour cells had a tendency to increase after PST (*P*=0.098). In the subgroup treated with AC regimen, grades of ALDH1+ tumour cells increased after PST with a marginal significance (*P*=0.059) (Figures 2C–F). Also, we investigated whether the changes of putative CSC population after PST varied according to breast cancer subtype. There were increasing trends of the putative CSC populations after PST regardless of the breast cancer subtype, and especially, grades of ALDH1+ tumour cells were significantly increased after PST in HER2+ subtype (*P*=0.034) (Table 3).

We analysed the association between the changes of CD44+/CD24− and ALDH1+ tumour cell population and clinicopathologic characteristics of tumour after PST. The cases with increased CD44+/CD24− tumour cell populations after PST showed high Ki-67 proliferation index in post-chemotherapy specimens (*P*=0.038) and tended to show high post-neoadjuvant therapy pathologic T (ypT) stage (*P*=0.091) and ER negativity (*P*=0.067). The cases with increased ALDH1+ tumour cell population after PST were associated with ER negativity (*P*=0.017) and p53 overexpression (*P*=0.030) (Table 4).

**Figure 4:**
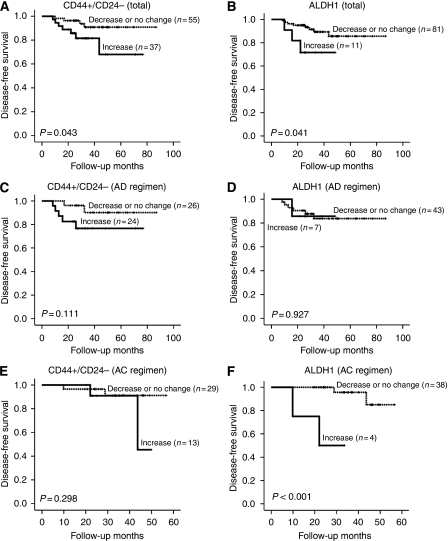
Disease-free survivals according to changes of the putative CSC population after primary systemic therapy. Patients with increased proportions of CD44+/CD24− (**A**) and ALDH1+ (**B**) tumour cells showed significantly poorer DFS than the other patients in total. In the subgroup analyses by primary systemic therapy (PST) regimen (AD or AC regimen), the patients with the increase of CD44+/CD24− tumour cell populations tended to have shorter DFS times than the remaining patients irrespective of PST regimen (**C** and **E**). With regard to the changes of ALDH1+ tumour cell populations in the subgroup analyses by PST regimen (**D** and **F**), the survival difference was much greater in the subgroup receiving AC regimen (**F**), but not in the subgroup treated by AD regimen (**D**). AD=doxorubicin plus docetaxel; AC=doxorubicin plus cyclophosphamide.

We also investigated the relationships of the changes of CD44+/CD24− and ALDH1+ tumour cell population after PST with DFS. At the time of the analysis, the median follow-up was 3 years (range, 1–7 years). There were four (4%) loco-regional recurrences as first events and seven (8%) distant metastases. In Kaplan–Meier survival analyses, the patients with increased CD44+/CD24− and ALDH1+ tumour cell populations had a significantly shorter DFS times than the remaining patients (*P*=0.043 and 0.041, respectively) (Figures 4A and B). Subgroup analyses by the PST regimen revealed that the survival difference was much greater in the patients receiving AC regimen between the group with increased ALDH+ tumour cell population after PST and the remaining group (*P*<0.001). On the other hand, there was no survival difference between the two groups in patients treated with AD regimen. With regard to the changes of CD44+/CD24− tumour cell populations, the patients with the increase of CD44+/CD24− tumour cell populations tended to have shorter DFS times than the remaining patients irrespective of PST regimen, although they were not statistically significant (Figures 4C–F). In multivariate analysis, ypT stage (ypT0-2 *vs* ypT3-4; HR, 4.82; 95% CI: 1.28–18.18; *P*=0.020) was identified as an independent predictor for DFS. However, the changes of CD44+/CD24− and ALDH1+ tumour cell populations did not remain as independent prognostic factors.

## Discussion

The present study showed that putative CSCs identified by CD44/CD24 and ALDH1 immunohistochemistry significantly increased after PST in human breast cancer tissue. In addition, the cases with increased CD44+/CD24− tumour cell populations after PST were found to have high Ki-67 proliferation index in post-chemotherapy specimens. More importantly, we report for the first time that the patients in whom the putative CSC population increased after PST had significantly shorter DFS times than patients in whom the putative CSC population were not changed or decreased. Furthermore, in the subgroup receiving AC regimen, the DFS time was much shorter in patients with increased ALDH+ tumour cell population after PST than in the remaining patients. These findings indicate that putative CSCs with CD44+/CD24− or ALDH1+ phenotypes have an important role in chemoresistance to conventional AD and AC therapy and in disease progression.

There are several previous reports that CSCs contributes to chemoresistance in cancer therapeutics. However, most of them are *in vitro* studies (Liu *et al*, 2006; Ghods *et al*, 2007; Yu *et al*, 2007; Fillmore and Kuperwasser, 2008), and there are few reports showing an association between CSCs and chemoresistance in human breast cancer tissue. Some previous comparative studies using paired human breast cancer tissue obtained before and after chemotherapy found that CD44+/CD24− (Li *et al*, 2008) or ALDH1+ (Tanei *et al*, 2009) tumour cells increased significantly after chemotherapy, which is in agreement with our results. However, other workers detected no significant enrichment of ALDH1+ tumour cells (Resetkova *et al*, 2010) or even a reduction of CD44+/CD24− tumour cells (Aulmann *et al*, 2010) after PST. The contradictory results could be explained as follows. First, the PST regimens and cycles differed in the various studies, and there may be treatment-specific influences on outcomes. Alternatively, differences in immunostaining methods and scoring protocols may have contributed to the discrepancies. *In vivo* studies using larger cohorts and more standardised methods will be required to settle the matter.

We characterised the putative CSC-rich breast cancer by assessing the relationship between CD44/CD24 or ALDH1 expression in pre-chemotherapy tissue and clinicopathologic parameters. In the current study, most of CD44+/CD24− or ALDH1+ cell-rich tumours belonged to the basal-like subtype, which accords with the several previous reports pointing to a relation between CSCs and the basal-like subtype (Honeth *et al*, 2008; Morimoto *et al*, 2009; Nalwoga *et al*, 2010; Park *et al*, 2010). These tumours displayed features of aggressive tumours, such as high histologic grade and high Ki-67 proliferation index, which are also characteristics of basal-like breast cancers as well.

With regard to the effect of CSC population size on patients’ survival, there have been contradictory results. Whereas some studies failed to find a relationships between them (Abraham *et al*, 2005; Resetkova *et al*, 2010), others showed an inverse correlation (Ginestier *et al*, 2007; Morimoto *et al*, 2009; Charafe-Jauffret *et al*, 2010). In the present study, we found that increased CD44+/CD24− and ALDH1+ tumour cell populations in post-chemotherapy specimen were prognostic factors for DFS, despite the fact that the size of the CD44+/CD24− or ALDH1+ tumour cell populations in pre-chemotherapy biopsy were not related to patients’ survival. The CD44+/CD24− and ALDH1+ tumour cells in post-chemotherapy specimens may constitute more specific chemoresistant CSC populations than those in pre-chemotherapy specimens, and thus may have greater clinical significance. However, we cannot rule out the possibility that differentiated tumour cells acquire the CSC phenotype after chemotherapy due to selection pressure. In respect to breast cancer subtypes on patients’ survival after PST, triple negative or basal-like breast cancers have been reported to show worse survival due to high relapse among those with residual disease after PST, despite higher chemosensitivity to conventional anthracyclin-based chemotherapy (so-called ‘triple-negative paradox’) (Carey *et al*, 2007; Bhargava *et al*, 2010). As basal-like tumours are enriched with CD44+/CD24− or ALDH1+ tumour cell population, as shown in this study, one may expect that the cases with increased CSC population after PST were mostly chemoresistant basal-like subtype and thus, show wore prognosis. However, there were no differences in the number of cases with increased CSC populations according to subtype. And even if patients’ survival was analysed only in the non-pCR group, we can find the tendency to have worse survival in cases with increased CD44+/CD24− or ALDH1+ tumour cell populations (*P*=0.127, 0.087, respectively; data not shown).

Although this study revealed prognostic significance of increased putative CSC population after PST in total, we failed to show its prognostic significance in the subgroup analyses according to PST regimen except for the changes of ALDH1+ tumour cell population in the subgroup receiving AC regimen. However, as our study was limited to a short follow-up period and a small number of cases, more extensive studies are needed to confirm the prognostic value of the CSC populations in post-chemotherapy specimens, especially, according to different chemotherapeutic regimens.

We found that ALDH1 positivity was significantly associated with the likelihood of pCR in breast cancer. Also, the group of patients showing pCR had a higher proportion of CD44+/CD24− tumour cells than those not showing pCR, although the difference was not statistically significant. These observations would seem to indicate that CSCs are chemosensitive, which appears to contradict our suggestion that they have a key role in chemoresistance. However, if cellular heterogeneity exist within the CSC populations detected by the putative breast CSC markers, all the CD44+/CD24− and ALDH1+ tumour cells would not necessarily be CSCs. Accordingly, those that were easily killed by PST in this study are not likely to be CSCs *per se*, but rather transit amplifying cells. Other important points to note are that breast cancers that have high histologic grades, are ER negative and have high Ki-67 proliferation indices, are prone to achieve pCR following PST, as demonstrated in many previous studies (Ellis *et al*, 1998; Chang *et al*, 1999; Rouzier *et al*, 2005; Carey *et al*, 2007) and that ER-negative cancers, especially basal-like breast cancers, are enriched in CSCs (Honeth *et al*, 2008; Morimoto *et al*, 2009; Nalwoga *et al*, 2010; Park *et al*, 2010). Taken together, the reason why tumours with high CD44+/CD24− and ALDH1+ cell populations tend to achieve pCR may be related to their possession of pathologic features predictive of pCR (high histologic grade, ER negativity and high Ki-67 proliferation index, etc.), rather than with their stem cell properties.

The CSC hypothesis has important clinical implications for early detection, prevention and treatment of breast cancer. On the basis of that hypothesis, clinical trials are now being initiated that use drugs targeting CSCs or CSC-regulatory molecules, such as *γ* secretase inhibitors that are able to inhibit Notch signalling and inhibitors of hedgehog signalling (Kakarala and Wicha, 2008). Our observation of the association between CSCs and chemoresistance in the present study underlines the importance of targeting therapy to CSCs.

In conclusion, the present study indicates the chemoresistant property of putative CSCs to the conventional anthracyclin-based chemotherapy and their role in disease progression by demonstrating an increase in the proportion of CSCs after PST and their association with reduced DFS. These findings emphasise the need to target CSCs in breast cancer treatment. We also suggest that the breast CSC population detected by CD44/CD24 or ALDH1 expression is heterogeneous and hence that validation of these CSC markers and the development of the more definitive markers would be important.

## Figures and Tables

**Table 1:** Association between pre-chemotherapy CD44+/CD24− tumour cell proportions and ALDH1 positivity and clinicopathologic characteristics of the tumours

	**CD44+/CD24− tumour cell population**		**ALDH1 expression of tumour cells**		
**Characteristics**	**Median (IQR) (%)**	***P*-value** a	**Negative (no. (%))**	**Positive (no. (%))**	***P*-value** b
*cT stage*		0.053			0.159
T1 (2) and T2 (47)	5 (1–42.5)		40 (50)	9 (75)	
T3 (30)	5 (1–22.5)		28 (35)	2 (17)	
T4 (13)	45 (5–77.5)		12 (15)	1 (8)	
*cN stage*		0.265			1.000
N0 (12)	25 (1–63.8)		11 (14)	1 (8)	
N1–3 (80)	5 (1–40)		69 (86)	11 (92)	
*cTNM stage*		0.288			0.538
II (41)	5 (1–35)		37 (46)	4 (33)	
III (51)	10 (1–45)		43 (54)	8 (67)	
*Histologic grade*		0.002			0.007
Grade I (7)	5 (1–50)		7 (9)	0(0)	
Grade II (45)	5 (0–15)		43 (56)	2 (17)	
Grade III (37)	20 (5–65)		27 (35)	10 (83)	
*Oestrogen receptor*		<0.001			<0.001
Negative (35)	20 (5–65)		24 (30)	11 (92)	
Positive (57)	5 (0–20)		56 (70)	1 (8)	
*HER2*		0.134			0.746
Negative (63)	10 (1–55)		54 (68)	9 (75)	
Positive (29)	5 (1–15)		26 (32)	3 (25)	
*Breast cancer subtype*		0.001			<0.001
Luminal A (40)	5 (0–35)		40 (50)	0 (0)	
Luminal B (16)	3.5 (0–15)		15 (19)	1 (8)	
HER2+ (13)	10 (1.5–20)		11 (14)	2 (17)	
Basal-like (23)	45 (15–70)		14 (17)	9 (75)	
*Ki-67 labelling index*		0.064			0.020
⩽20% (44)	5 (0–35)		42 (53)	2 (17)	
>20% (48)	12.5 (2–52.5)		38 (47)	10 (83)	
*P53 staining*		0.124			0.856
Negative (67)	10 (1–45)		58 (73)	9 (75)	
Positive (25)	2 (1–15)		22 (27)	3 (25)	

Abbreviations: ALDH1=aldehyde dehydrogenase 1; cN=clinical node; cT=clinical tumor; cTNM=clinical tumor-node-metastasis; HER2=human epidermal growth factor receptor 2; IQR=interquartile range.

a Mann–Whitney *U-*test or Kruskal–Wallis test.

b *χ*^2^ or Fisher's exact test.

IQR (25–75%).

**Table 2:** Univariate and multivariate logistic regression models for predictors of pCR following PST

**Variables**	**Odds ratio**	**95% CI**	***P*-value**
*Univariate analysis*			
CD44+/CD24− tumour cell proportion (continuous)	1.014	0.996–1.034	0.135
ALDH1 positivity (negative *vs* positive)	6.429	1.649–25.056	0.007
Histologic grade (grades I, II *vs* III)	4.295	1.087–13.689	0.037
Oestrogen receptor (ER+ *vs* ER−)	2.125	0.650–6.942	0.212
Breast cancer subtype (non basal-like *vs* basal-like)	3.126	0.927–10.539	0.066
cT stage (cT3, 4 *vs* cT1, 2)	3.419	0.879–13.367	0.077
cN stage (cN1–3 *vs* cN0)	1.255	0.242–6.507	0.787
PST regimen (AD *vs* AC)	2.118	0.636–7.051	0.221
			
*Multivariate analysis*			
ALDH1 positivity (negative *vs* positive)	4.150	0.970–17.765	0.055
Histologic grade (grades I, II *vs* III)	2.625	0.670–10.276	0.166

Abbreviations: AC=doxorubicin plus cyclophosphamide; AD=doxorubicin plus docetaxel; ALDH1=aldehyde dehydrogenase 1; CI=confidence interval; cN=clinical node; cT=clinical tumor; pCR=pathologic complete response; PST=primary systemic therapy.

**Table 3:** Changes of putative CSC population before and after PST according to the breast cancer subtype in the cases not achieving pCR

**Breast cancer subtypes**	**CD44+/CD24− tumour cell proportion (median (IQR), %)** a		**Changes of ALDH1 grade (case no. with increase/no change/ decrease of ALDH1 grade after PST)** a
	**Before PST**	**After PST**	
Luminal A (*n*=38)	5 (0–35)	6.5 (0–47.5)	2/36/0
Luminal B (*n*=13)	3.5 (0–15)	0 (0–22.5)	1/12/0
HER2+ (*n*=11)	10 (1.5–20)	40 (0.5–70)	5/6/0^b^
Basal-like (*n*=17)	45 (15–70)	55 (20–80)	3/13/1

Abbreviations: ALDH1=aldehyde dehydrogenase 1; CSC=cancer stem cell; HER2=human epidermal growth factor receptor 2; IQR=interquartile range; pCR=pathologic complete response; PST=primary systemic therapy.

a Wilcoxon's signed-rank test;

b *P*=0.034.

**Table 4:** Association between the changes of putative CSC population after PST and clinicopathologic characteristics of the tumours in the post-chemotherapy specimens

	**CD44+/CD24− tumour cell proportion**			**ALDH1 grade**		
**Characteristics**	**No change or decrease (no. (%))**	**Increase (no. (%))**	***P*-value** a	**No change or decrease (no. (%))**	**Increase (no. (%))**	***P*-value** a
*ypT stage*			0.091			0.185
T0–2 (79)	50 (91)	29 (78)		71 (88)	8 (73)	
T3–4 (13)	5 (9)	8 (22)		10 (12)	3 (27)	
*ypN stage*			0.267			0.745
N0 (31)	21 (38)	10 (27)		28 (35)	3 (27)	
N1–3 (61)	34 (62)	27 (73)		53 (65)	8 (73)	
*Oestrogen receptor* b			0.067			0.017
Negative (30)	12 (29)	18 (49)		22 (32)	8 (73)	
Positive (49)	30 (71)	19 (51)		46 (68)	3 (27)	
*HER2^c^*			0.933			0.150
Negative (58)	31 (74)	27 (73)		52 (77)	6 (55)	
Positive (21)	11 (26)	10 (27)		16 (23)	5 (45)	
*Ki-67 labelling index*			0.038			0.311
⩽20% (54)	33 (79)	21 (57)		48 (71)	6 (55)	
>20% (25)	9 (21)	16 (43)		20 (29)	5 (45)	
*P53 staining*			0.575			0.030
Negative (64)	35 (83)	29 (78)		58 (85)	6 (55)	
Positive (15)	7 (17)	8 (22)		10 (15)	5 (45)	

Abbreviations: ALDH1=aldehyde dehydrogenase 1; CSC=cancer stem cell; HER2=human epidermal growth factor receptor 2; PST=primary systemic therapy; ypT=post-neoadjuvant therapy pathologic tumor; ypN=post-neoadjuvant therapy pathologic node.

a *χ*^2^ or Fisher's exact test.

b Oestrogen receptor status was changed from positive to negative after PST in two cases.

c HER2 status was converted from negative to positive after PST in a case.
